# Supplementary material for: Understanding water behaviour on 2D material interfaces through single-molecule motion on h-BN and graphene
Source: Nat Commun. 2025 Nov 25;16:10465. doi: 10.1038/s41467-025-65452-1 (PMC12647898; doi:10.1038/s41467-025-65452-1)
Supplement: Supplementary file 1 — Supplementary Information [file 41467_2025_65452_MOESM1_ESM.pdf]

Supplementary Information for
Understanding water behaviour on 2D material interfaces through single-molecule motion on h-BN and graphene

Supplementary Note 1: Additional experimental details

1.1 Sample preparation

The Ni single crystal has been cleaned by multiple cycles of Ar^+ sputtering and annealing to 1050 K. The hexagonal boron nitride layer was prepared by chemical vapour deposition (CVD) according to the procedure given by Auwärter *et al.*, where the Ni(111) surface was maintained at 1050 K and exposed to the gas-phase precursor borazine ($B_3H_6N_3$). The hot Ni surface acts as a catalyst, initiating the chemical reactions such as the breaking of the borazine rings, dehydrogenation of borazine, and subsequent formation of a commensurate, epitaxial overlayer due to the small lattice mismatch between Ni(111) and h-BN.[1,2] During the whole CVD growth, the He intensity of the first-order diffraction peak has been recorded. As soon as the borazine is converted to h-BN, the intensity increases until it saturates at a value above the initial intensity. At this point, a complete overlayer is formed, and the dosing of borazine is stopped. The growth process itself is relatively slow and proceeds over a time scale of a few hours.
The sample temperature was measured with a chromel-alumel thermocouple, providing relative temperature values with a precision of ± 0.1, K. Investigations of water adsorption and desorption processes were conducted by dosing from a microcapillary array beam doser, employing a motorised leak valve which offers precise control over water pressure. The leak valve, managed by a feedback control system, ensured the maintenance of a constant pressure throughout the experiments. The doser received water from a baked stainless steel tube filled with de-ionised water, with the vapour pressure generated by the liquid phase at room temperature for water supply.
Prior to every series of adsorption, water was re-purified using a process of several freeze-pump-thaw cycles, where the water inside the tube was frozen, and the gas phase above the frozen ice was pumped away.

1.2 Coverage calibration

All measurements were consistently conducted at a coverage range Θ between 0.12 ML and 0.20 ML. In our experiments, we controlled the coverage by taking the helium reflectivity of the surface into account. As soon as the water is deposited onto the surface, the reflectivity drops rapidly. At an attenuation of roughly $I/I_0 = 0.25$, we stopped dosing. In Supplementary Figure **1**, the uptake behaviour of water is shown for $T = 120$ K and $T = 130$ K. From these uptake curves, the coverage can be estimated by applying the Langmuir model, which relates the surface coverage to the exposure through the kinetic theory of gases.[3]
Our dynamical measurements at 120 K were performed at exposures between 0.47 L and 0.75 L, verified by regularly checking the reflectivity between the single ISFs.
Assuming a constant sticking coefficient of unity (see Refs.[4–6]) and with a monolayer coverage corresponding to $n = 0.115$ molecules$\cdot$Å^{-2} $= 0.618$[5], a gas temperature of $T = 290$ K of the water vapour and the mass of the water molecule $m = 18.0153\,\mathrm{g\,mol^{-1}}$, the coverage of our measurements at $T = 120$ K is estimated to be between 0.12 ML and 0.20 ML.
The coverage calibration closely follows the approach described in Ref.[7] and its Supplementary Information. There, water adsorption on graphene/Ni allowed for two independent coverage calibration methods: one based on dosing rate and helium reflectivity decay, and a second based on the analysis of dephasing rates via de Gennes narrowing, reflecting repulsive interactions in the collective diffusion regime. Both methods yielded consistent estimates, validating the cross section and calibration procedure. Specifically, the impingement rate was derived from the measured partial pressure of water in the chamber, corrected for the microcapillary flux enhancement.[8] Here we employ the same scattering cross $\Sigma = (120 \pm 20)$ Å^2 as in[7] consistent with literature values in Ref.[9]) with the calculated coverage following a "stick and sit" adsorption model at low temperatures.[10]

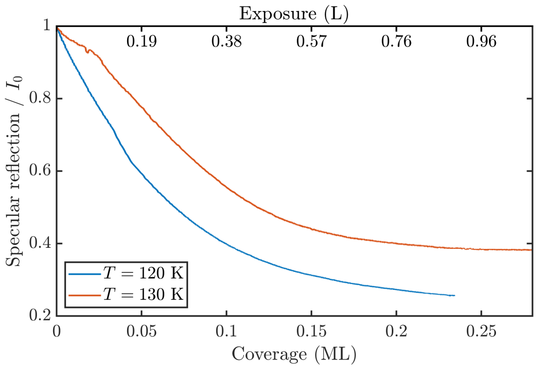

Supplementary Figure 1: **Water adsorption on the h-BN surface right before the dynamical measurements** at 120 K and 130 K. The normalised specular reflection is plotted against the coverage (bottom axis) and exposure (top axis).

The reflectivity behaviour in Supplementary Figure **1** is mostly consistent with random adsorption of non-interacting scatterers.[10] In this model, for randomly distributed adsorbates with large effective scattering cross sections that overlap, the specular attenuation appears linear when plotted on a logarithmic scale.[10,11] The normalised specular intensity is then related to the adsorbate coverage Θ via the expression

$$I/I_0 = (1 - \Theta)^{n_s \Sigma / \cos \vartheta_i},$$

where n_s is the surface site density, Σ the effective scattering cross section, and ϑ_i the incident angle of the helium beam. This form approximates an exponential decay for low to moderate coverages and confirms the independence of scattering centres with a constant cross section.
In contrast, the curve for $T = 130$ K exhibits an initial deviation from this trend: an approximately linear decay in intensity is observed directly on a linear scale over the initial exposure range (up to approximately 0.03 ML), followed by a transition to exponential-like attenuation. While this behaviour may suggest increased water mobility and potential attractive interactions or 2D island nucleation at elevated temperatures, we refrain from overinterpreting the deviation in the uptake curve, given that the reflectivity returns to an exponential form.
In summary, the determined uncertainty is primarily due to limitations in doser calibration and geometry as well as some inevitable variation throughout the duration of the dynamics measurements,

particularly at the final coverage.

1.3 Water adsorption and desorption

We have carried out isothermal adsorption measurements of H_2O on h-BN/Ni(111) surface with the HeSE spectrometer monitoring the specular reflected signal during water deposition. Dosing water at 120 K results in the growth of a water multilayer, covering the entire h-BN surface. Since the water molecules scatter the He atoms diffusively, the reflectivity decays rapidly (see Supplementary Figure **2**, which is typical of disordered structures. Slightly heating the sample to $T = 125\,\mathrm{K}$ after about 17 minutes (not shown here) did not impact the helium reflectivity, and hence, the surface remained unchanged. During the whole measurement, the dosing valve remained open.

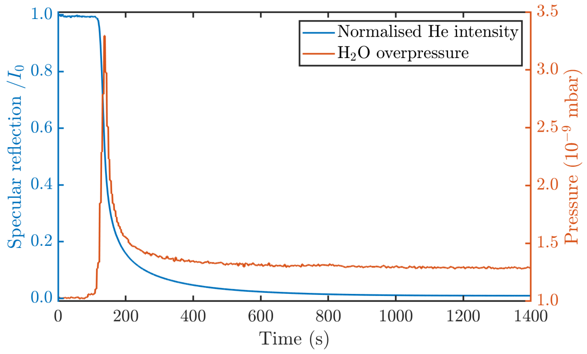

Supplementary Figure 2: **Uptake curve for water dosing on h-BN.** Specular helium reflectivity (red curve) during uptake of water on the h-BN surface at $T = 120\,\mathrm{K}$. The intensity drops rapidly to zero since individual water molecules obscure the underlying h-BN, indicating the growth of an amorphous ice layer. The red curve displays the pressure in the main chamber during the deposition of H_2O.

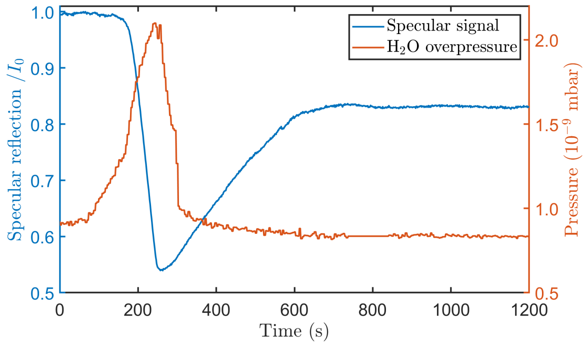

Supplementary Figure 3: **De-wetting and island formation for isothermal H_2O uptake on h-BN at 135 K.** The He specular reflection is monitored during water deposition onto the h-BN surface at constant temperature. Closing the dosing valve after about 6 minutes leads to a recovery of the reflectivity, indicating a partial de-wetting of the surface.

A similar uptake measurements at $T = 135\,\mathrm{K}$ is shown in Supplementary Figure **3**. Again, water deposition on the h-BN surface results in a fast decay of the helium specular signal. After about 6 minutes, when the helium reflectivity had fallen to half its initial value, the valve of the dosing arm was closed and the dosing stopped. The helium signal recovers immediately, but not to its initial value. This result suggests that water has not desorbed, but rather has migrated to form isolated islands, resulting in partial dewetting of the surface to reveal pristine h-BN, which is a different behaviour than at $T = 120\,\mathrm{K}$ (see Supplementary Figure **2**), where the growth of an amorphous ice layer was observed.

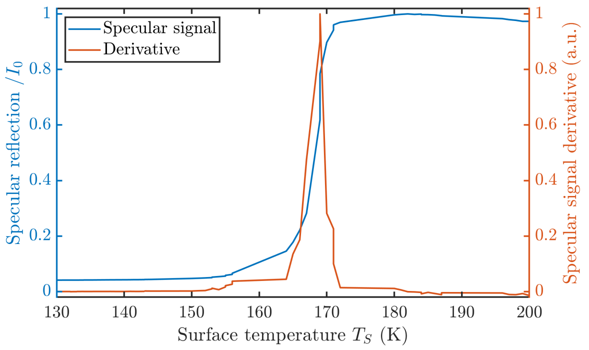

Supplementary Figure 4: **Thermal desorption measurement for multilayer H_2O/h-BN/Ni(111) prepared at low temperature**. The specular signal is monitored while heating the surface at a constant rate. Applying the Readhead equation gives a desorption energy of 530 meV. The desorption temperature of the main peak is equivalent to desorption from bulk ice.

We have also performed a thermal desorption measurement by increasing the temperature and simultaneously measuring the He reflectivity of the surface that has initially been covered with water at $T = 130\,\mathrm{K}$. In Supplementary Figure **4**, the specular signal and its derivative are plotted against the surface temperature. A widely used model for analysing temperature-programmed desorption (TPD) spectra is the Redhead formalism, which assumes first-order kinetics and provides an analytical expression for the desorption energy E_d [12]:

$$E_\mathrm{d} = \mathrm{k_B} T_\mathrm{d} \left[\ln \left(\frac{\nu T_\mathrm{d}}{\beta} \right) - 3.64 \right],$$

where β denotes the linear heating rate, ν is the pre-exponential factor, and T_d is the temperature corresponding to the maximum desorption rate, identified from the peak in the derivative of the helium signal. Although the model is approximate, it remains widely employed, for example, in the analysis of water desorption from highly oriented pyrolytic graphite (HOPG).[13] Using $T_\mathrm{d} = (169 \pm 5)\,\mathrm{K}$, $\beta = 0.76\,\mathrm{K\,s^{-1}}$, and the estimated desorption energy is:

$$E_\mathrm{d}^{(1)} = (580 \pm 20)\,\mathrm{meV}.$$

Nevertheless, it is well established that for water on weakly interacting surfaces, such as graphene and related systems, zero-order desorption kinetics are more commonly observed, particularly at coverages exceeding a single monolayer. This behaviour arises from the formation of hydrogen-bonded water clusters, which decouple the desorption kinetics from the surface coverage.[14,15] To account for this, the desorption energy was additionally estimated assuming zero-order kinetics by numerically inverting the Polanyi–Wigner equation under linear heating. In this formulation, the pre-exponential factor ν is effectively coverage-dependent (i.e., $\nu = \nu_0 \theta_0$), and the relation between E_d, T_d, β, and ν lacks a closed-form expression. Employing the same experimental parameters and solving numerically yields:

$$E_\mathrm{d}^{(0)} = (530 \pm 60)\,\mathrm{meV},$$

with the increased uncertainty reflecting the combined limitations of the experimental approach, coverage estimation, and kinetic model assumptions. These values provide a reference for the desorption temperature regime of water on h-BN and represent, to the best of our knowledge, the first such measurement for this

system. Interestingly, our value is similar to the desorption energy of water on graphene[7] and close to the sublimation enthalpy of ice.[5,6]

1.4 Additional dynamics measurements

Similar to our dynamical measurements in $\overline{\Gamma M}$ direction (see main text Fig. 4), we also recorded ISFs with the sample aligned along the other high-symmetry orientation $\overline{\Gamma K}$, both at $T = 120\,\mathrm{K}$ and $T = 130\,\mathrm{K}$. Although having only a few data points, Supplementary Figure **5** illustrates that α exhibits a similar behaviour along the $\overline{\Gamma K}$ azimuth compared to $\overline{\Gamma M}$, e.g. α does not go to zero for ΔK approaching zero.

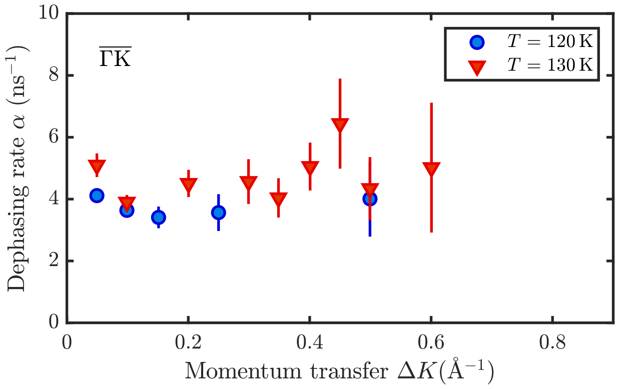

Supplementary Figure 5: Additional dephasing rate $\alpha(\Delta K)$ measurements for ≈ 0.2 ML H_2O along $\overline{\Gamma K}$. Momentum transfer dependence of the dephasing rate $\alpha(\Delta K)$ along $\overline{\Gamma K}$ direction at 120 and 130 K, respectively. The error bars correspond to the confidence bounds (1σ) of the exponential fit (see also Fig. 4 in the main text).

Supplementary Note 2: Additional calculations

2.5 Details on dispersion corrected DFT

The determination of the global minimum for water adsorption on free-standing h-BN requires the exploration of the potential energy surface (PES). The water molecule underwent relaxation at high-symmetry positions across the h-BN surface, considering a range of initial orientations. Subsequently, the resulting adsorption energies were evaluated. The global minimum, depicted in Supplementary Figure **6**, exhibits an E_{ads} of $-0.188\,\mathrm{eV}$. Notably, this configuration closely resembles that observed for h-BN/Ni (Fig. 1 in the main text), featuring one hydrogen atom orientated downward directly toward the surface nitrogen. However, in this instance, the oxygen atom is positioned nearly directly above the nitrogen atom, while the second hydrogen atom extends across the surface toward an adjacent nitrogen atom.

2.6 The motion of dimers

Supplementary Figure **7** presents the calculated energy barriers associated with the translational and rotational motion of water dimers adsorbed on the h-BN/Ni surface. In contrast to the relatively facile motion observed for single water molecules (cf. Fig. 3 in the main text), the energy barriers for dimer mobility are substantially higher. Specifically, the barriers for translational diffusion along different lattice directions are 79 meV and 99 meV, while rotational reorientation, which requires breaking and reforming the intermolecular hydrogen bond, exhibits an even

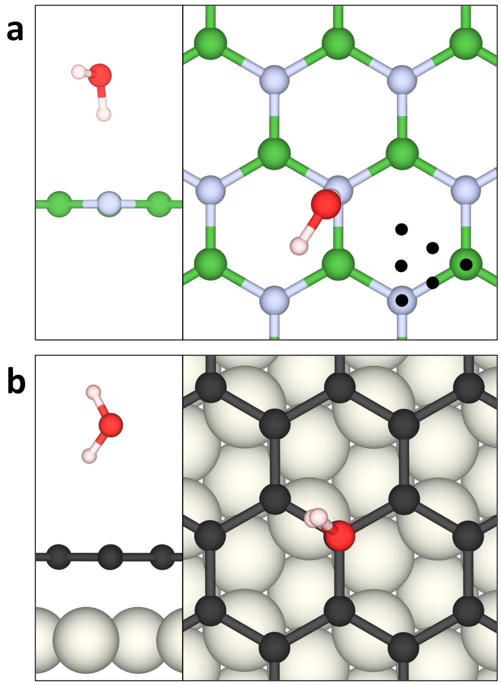

Supplementary Figure 6: Water adsorption sites on free-standing h-BN and graphene/Ni(111). a The global minimum position of water on h-BN with an E_{ads} of $-0.19\,\mathrm{eV}$. The initial surface sites sampled to establish the PES are shown as black dots. **b** The global minimum position of water on graphene/Ni(111) with an E_{ads} of $-0.21\,\mathrm{eV}$.

higher barrier of 220 meV. These values exceed the experimentally determined activation energy for single-molecule diffusion (24 meV) by factors of approximately 3-9, indicating that dimers are far less mobile under the experimental temperature range of 120-140 K.

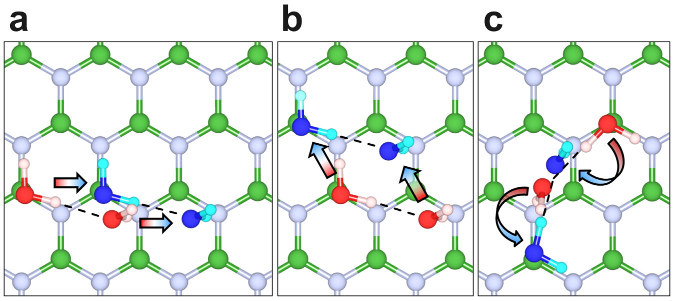

Supplementary Figure 7: Diffusion of water dimers on hexagonal boron nitride. a and **b** show diffusion by translation with energy barriers of 79 meV and 99 meV, respectively. **c** shows the rotation of water dimers, which rotate and break the hydrogen bond with a barrier of 220 meV before reforming a hydrogen bond in the opposite direction.

To further explore the potential for dimer dissociation, we computed the minimum energy pathway for the separation of a water dimer on h-BN/Ni(111), as shown in Supplementary Figure **8**. The dissociation process proceeds via a coupled rotation-translation mechanism and is associated with a substantial energy barrier of 220 meV. This implies that, once formed, dimers are kinetically stabilised and unlikely to dissociate spontaneously at the current experimental temperatures.
Although the formation of dimers is thermodynamically allowed, especially under low but finite coverages where water-water interactions become possible, their significantly reduced mobility

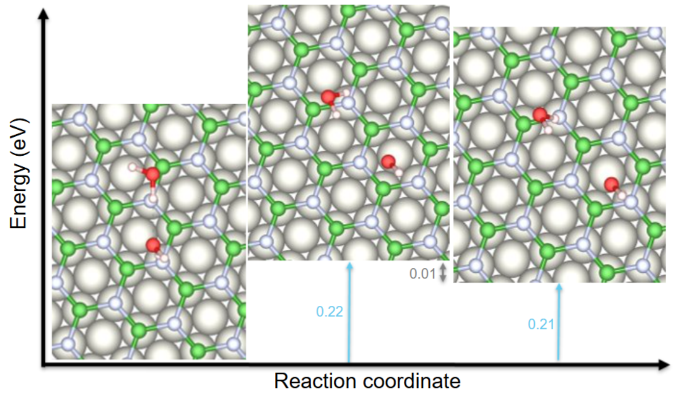

Supplementary Figure 8: **Reaction pathway for the dissociation of a water dimer on h-BN/Ni(111).** The reaction proceeds via a rotation of the dimer coupled with translation to separate the pair, overcoming an energy barrier of 220 meV.

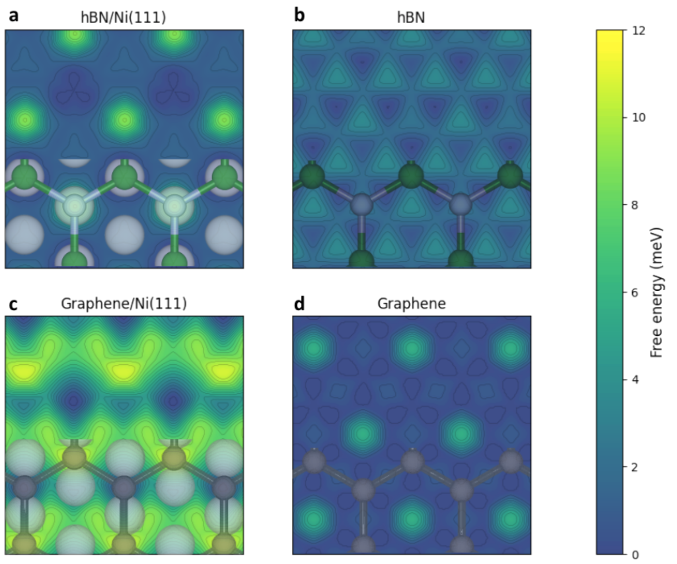

Supplementary Figure 9: **Comparison of the water adsorption free energy profiles.** **a** h-BN/Ni(111), **b** h-BN, **c** graphene/Ni(111), **d** graphene. **a**, **b** and **d** show a more uniform and connected distribution of free energies, whereas **c** indicates a more localised distribution favouring the C atom site (Source data are provided as a Source Data file).

means they do not contribute appreciably to the observed dynamical behaviour. In our experimental regime, which corresponds to submonolayer (low-coverage) conditions, water molecules are predominantly isolated and spatially separated, further limiting the frequency of dimer formation. Therefore, while dimers may exist transiently, their high diffusion barriers preclude them from participating in the quasi-continuous motion detected by helium spin-echo spectroscopy. The observed dynamics are thus most accurately captured by focusing on single-molecule trajectories.

2.7 Ab initio molecular dynamics simulations

For the AIMD simulations of water on h-BN, the global minimum served as the initial geometry, and equivalent starting positions were employed for the graphene analogues.
To investigate variations in friction coefficients, we computed the free energy profile (ΔG) of the water molecule at different sites based on the probability distribution of the water molecule on various surface sites as defined by:

$$\Delta G = -\mathrm{k_B} T \ln\left(\frac{P_i}{P_0}\right) \quad , \tag{1}$$

where P_i is the probability of finding a water molecule above site i and P_0 is the probability associated with the most probable site, $\mathrm{k_B}$ is the Boltzmann constant, and T is the temperature of the system. Supplementary Figure **9** illustrates the ΔG distribution of the water molecule's presence at various surface sites. The first 100 timesteps were discarded to allow time for the molecule to dissociate from the starting site. In each time step, the centre of mass of the water molecule was determined, and the closest surface site was calculated, accounting for the dynamic movement of the underlying surface atoms. The data from all AIMD simulations on a specific system was aggregated. The probability of locating a water molecule at distinct points was found, and the ΔG values between points were estimated by interpolation.

For the h-BN/Ni system, our findings coincide with the preferred positions and the PES determined through the assessment of adsorption energies. The N site exhibits diminished favorability compared to the B site in the h-BN/Ni(111) system, while the region within the hole site is favoured, with only marginal differences in ΔG around the hole site. In contrast, the free-standing h-BN site reveals comparable ΔG values for both B and N sites, with an overall preference for the bridge sites. The distribution of ΔG exhibits greater uniformity in free-standing h-BN compared to h-BN/Ni(111). Both h-BN and h-BN/Ni(111) exhibit C_{3v} symmetry. In contrast, free-standing graphene, characterised by C_{6v} symmetry, avoids the centre of the hole site, demonstrating a uniform distribution around the C atoms and the hole site. Conversely, for the graphene/Ni(111) system, the ΔG values are far less uniform. It should be noted that the free-standing graphene C_{6v} symmetry is reduced to C_{2v} induced by the Ni substrate leading to two distinct carbon atom sites, one with a carbon atom adjacent to the FCC site (C^{FCC}) and the other with a carbon atom adjacent to the HCP site (C^{HCP}). The reduced symmetry leads to a discrepancy in the ΔG values, with the minimum being (C^{FCC})and (C^{HCP}) having a ΔG of $0.01\,\mathrm{eV}$. The E_{ads} for the carbon sites is $-0.213\,\mathrm{eV}$ and $-0.197\,\mathrm{eV}$ for the FCC adjacent and HCP adjacent sites, respectively, explaining the difference ΔG between the two sites. In contrast to interconnected potential energy surfaces (PES) observed in other systems, the movement of water on graphene/Ni involves overcoming substantial free energy barriers, leading to heightened friction on this surface.

In order to confirm the behaviour observed for the water monomer, we also conducted AIMD simulations for water dimers on free-standing graphene and h-BN. The friction values were consistently higher for the h-BN case compared to graphene, with a maximum ratio of 3.1. Therefore, our results suggest that, in our experiments, the diffusion of water on h-BN and graphene can be accurately represented by the motion of single water monomers. The Boltzmann distribution can then be applied to give the probability of each conformation as a function of temperature using: [16]

$$p_j = \frac{\exp\left(-\frac{\varepsilon_j}{\mathrm{k_B}T}\right)}{\sum_k \exp\left(-\frac{\varepsilon_k}{\mathrm{k_B}T}\right)} \quad , \tag{2}$$

Where p_j is the probability of conformation j of energy ε_j, $\mathrm{k_B}$ is the Boltzmann constant, T is the temperature of the system, and k indexes over all conformations sampled with energy ε_k. The probability of finding water at different surface sites on h-BN/Ni(111)

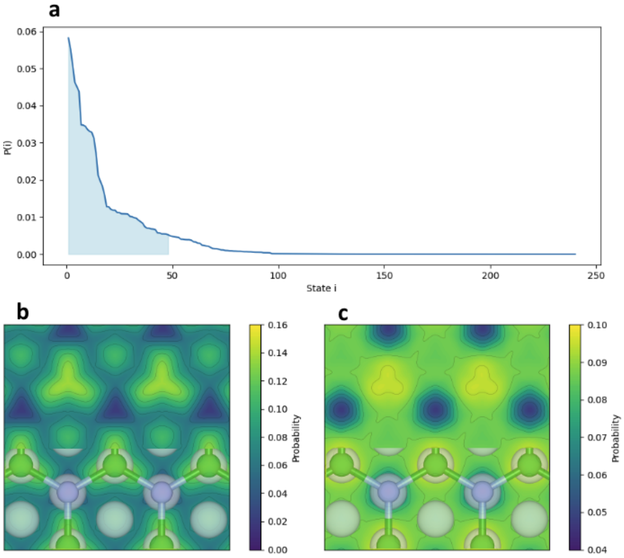

Supplementary Figure 10: **Boltzmann distribution and site probabilities of water adsorption on h-BN/Ni(111). a** Boltzmann distribution of water molecules in state i, the shaded area represents states that take up 90% of the probability distribution. The probability of finding water at a surface site (p_j) is calculated using the Boltzmann distribution in **b** and using the AIMD trajectories in **c** (Source data are provided as a Source Data file).

generated from the Boltzmann distribution and AIMD can be seen in Supplementary Figure **10**b and c, respectively. Constructing the Boltzmann distribution entailed exploring a configuration space by relaxing water conformations to pinpoint minima at specific sites. Conversely, in AIMD simulations, water molecules exhibit dynamic behaviour in response to external forces, leading to displacement from sampled sites and minima, thereby probing a wider range of sites and conformations. These differences result in the varying ranges evident in the probability distributions. Despite the variance in ranges displayed, both surfaces exhibit a comparable pattern, with the water molecule avoiding the N-site, favouring the B-site, and featuring large areas with a relatively uniform probability distribution. These similarities are due to the fundamental statistical behaviour dictating the behaviour of water molecules on the h-BN/Ni(111) surface realised through both AIMD and Boltzmann statistics.

2.8 Surface corrugation

2D materials have a variety of mechanisms that modify the atomic interactions and produce unique interfacial properties. One of these effects is surface corrugation, where there is a series of ordered parallel ridges or grooves on the surface of a material, forming a pattern of alternating peaks and valleys.
We explore the disorder of the surface by comparing the Root Mean Square Deviation (RMSD), a commonly used quantitative measure of the similarity between two superimposed atomic coordinates. We compare the RMSD of 2 ps of AIMD averaged over ten replicas to the "ordered" state - the same system but geometry optimised without the water. While the AIMD is conducted with a single water present, the water is removed during the RMSD analysis. Furthermore, collective shifts in the RMSD are removed during the analysis by minimising the overall rotation and translation of the system. In the analysis, we focus on the in-plane(x,y) RMSD and neglect the z components.
Comparing the RMSD of the optimised structure to the averaged

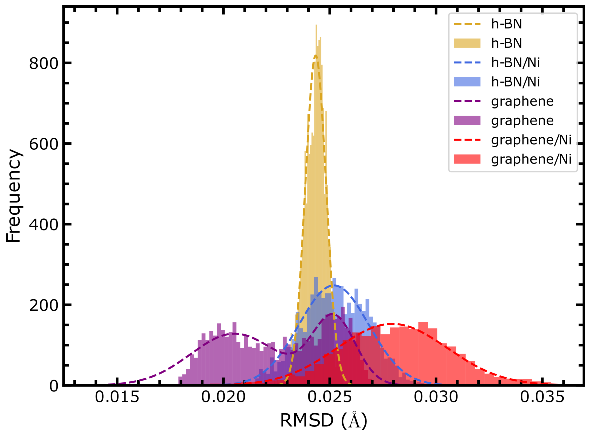

Supplementary Figure 11: **RMSD analysis of AIMD trajectories.** Comparing the RMSD of the optimised structure to the averaged AIMD replicas provides a single metric on the maximal overlap of the AIMD time series data. Here, the dynamic corrugation is shown as a function of the surface and the substrate. The results strongly correlate with the friction and indicate that the Ni substrate significantly impacts the dynamic corrugation and that graphene has the highest induced distortion, which leads to significantly higher friction values.

AIMD replicas provides a single metric on the maximal overlap of the AIMD time series data. Hence, a small RMSD value demonstrates a geometry similar to the optimised structure, whereas a large RMSD indicates a structure far from the optimised structure. Consequently, this provides a metric of dynamic corrugation.
We determine the following order of the RMSD (see Supplementary Figure **11**), representing the dynamic corrugation: graphene with a bimodal distribution but with the lowest RMSD, then h-BN, following this with only a minor difference h-BN/Ni, and finally graphene/Ni. The order exactly correlates with the friction value, indicating that the in-plane (x, y) RMSD could be used as a positive indicator of friction; a high RMSD indicates a significant distortion of the structure, which correlates with high friction.
The most notable change in RMSD is between graphene compared to the free-standing case vs. when supported by the Ni substrate. For the graphene/Ni case, the RMSD distribution peaks at 0.0279 Å with a broad standard deviation of 0.0026 Å. Whereas, for just graphene, the RMSD distribution has two peaks, one at a much lower value of 0.0205 Å with a standard deviation of 0.0021 Å.
On the other hand, for h-BN, the change induced by the Ni substrate is only a slight increase in the RMSD distribution peak location. However, the free-standing h-BN has a significantly tighter distribution, with a standard deviation of 0.0005 Å (vs 0.0016 Å). These results first indicate that the Ni substrate significantly impacts the dynamic corrugation and that graphene has the highest induced distortion, which leads to significantly higher friction values.

2.9 Details of ISF calculation from the AIMD

The trajectories obtained from the AIMDs can be used to calculate the (coherent and/or incoherent) ISF analytically.[17] For each water molecule j, the scattered amplitude as a function of the parallel momentum transfer $\Delta\mathbf{K}$ and the time step t is given by $A_j(\Delta\mathbf{K}, t) = \exp\left(-\mathrm{i}\Delta\mathbf{K}\cdot\mathbf{r}_j(t)\right)$, where $r(t)$ is the center of mass trajectory. Next, the scattering function from adsorbate i can be calculated as

$$S_j(\Delta\mathbf{K}, \Delta\omega) = \mathcal{F}[A_j]^* \cdot \mathcal{F}[A_j],$$

with $\mathcal{F}$ being the temporal Fourier transformation and $\hbar\omega$ the energy change. For incoherent scattering, the scattering function is the sum over all particles, $S_{inc} = \sum_j S_j$, whereas for the coherent case, the amplitudes of scattering from all particles, $A = \sum_j A_j$, are calculated first and then the scattering function can be computed as $S_{coh}(\Delta\mathbf{K}, \Delta\omega) = \mathcal{F}[A]^* \cdot \mathcal{F}[A]$. In the last step, the coherent and incoherent ISFs are calculated by inverse temporal Fourier transformation of S,

$$I(\Delta\mathbf{K}, t) = \mathcal{F}^{-1}\left[S(\Delta\mathbf{K}, \Delta\omega)\right].$$

For each simulation, the ISFs are calculated as above, and the final ISF is the mean over all simulation runs.

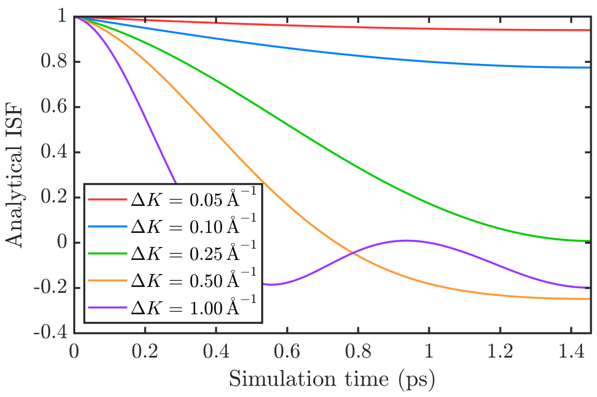

Supplementary Figure 12: ISFs calculated from the AIMD trajectories. Plot of the ISFs calculated from the AIMD trajectories following the approach described in Supplementary Note 2.5. It illustrates that the decay in the ISFs for ΔK approaching 0 remains greater than 0.

The ISFs calculated analytically for various values of ΔK from our AIMDs are shown in Supplementary Figure **12**. Even though a direct comparison to the experimentally measured ISFs is difficult, one can clearly see that the analytical ISFs exhibit a decay for ΔK (Supplementary Figure **12**) approaching zero, which confirms the interpretation that the $z-$component of the motion gives rise to the constant offset of $\alpha(\Delta K)$ (see main text Fig. 4.

2.10 Additional friction calculations

The error bar shown in Fig. 6a in the main text for the graphene/Ni(111) system illustrates the uncertainty in the computed friction coefficient $\lambda(t)$ when the simulation is extended beyond 2 ps. To assess the convergence and robustness of our friction estimates, we performed additional ab initio molecular dynamics (AIMD) simulations with identical parameters as those in the main text, increasing the simulation time to a minimum of 3 ps.

As shown in Supplementary Figure **13**, the friction coefficient increases from approximately $9.5 \cdot 10^7$ $\mathrm{Ns\,m^{-3}}$ at 2 ps to $9.7 \cdot 10^7$ $\mathrm{Ns\,m^{-3}}$ at 3 ps, corresponding to a 2% change. Although the gradient of $\lambda(t)$ remains slightly positive, the curve is subject to considerable statistical noise. These small variations indicate that the friction coefficient is approaching convergence, and we consider the 2 ps cutoff to provide a reliable estimate. This is also consistent with previous work by Tocci *et al.* [18], which demonstrated that a 2 ps sampling window is typically sufficient for obtaining converged friction values in similar systems. Importantly, the minor changes observed here are negligible compared to the overall differences between the four investigated systems, and thus do not affect the conclusions drawn from our comparative analysis.

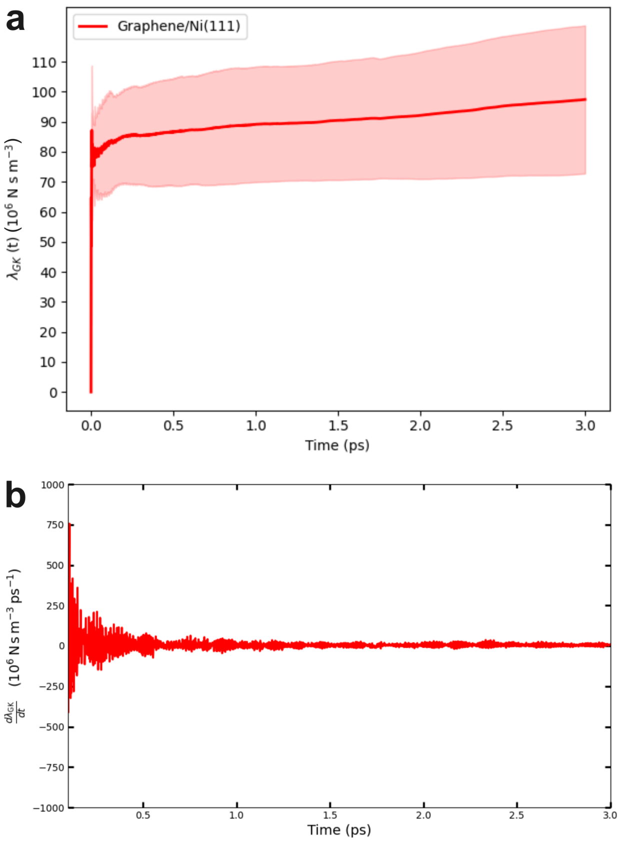

Supplementary Figure 13: Additional AIMD simulations for graphene/Ni(111). **a** Using identical parameters as in the main text and a minimum duration of 3 ps shows a corresponding increase of 2% of $\lambda(t)$ when continuing from 2 to 3 ps. **b** shows a plot of the gradient, where the first 0.1 ps of the derivative have been omitted.

2.11 Vibrational power spectrum

We investigate the vibrational power spectrum of intra- and intermolecular modes of water molecules and the surface by calculating the Fourier transform of the velocity autocorrelation function. We extract the velocity from the AIMD runs from CASTEP simulations. We then use the velocity of oxygen and hydrogen atoms in water molecules to estimate the vibrational power spectrum; the results are shown in Supplementary Figure **14** and Supplementary Figure **15**. The results in these figures are derived from the various AIMD trajectories of the h-BN, h-BN/Ni, graphene, and graphene/Ni systems. We repeat the AIMD from different starting conditions and compute an ensemble average of the power spectrum over the repeats. We employ a Hann window function for filtering to compensate for the finite truncation of the numeric fast Fourier transform. We normalise the spectrum such that the total integral is equal to unity, allowing a direct comparison of changes in the spectrum. Furthermore, the process is repeated to explore the case of isolated water, monomeric and water dimer diffusion on these surfaces to compare the systems within the same theoretical framework.

For the h-BN system, there are three leading vibrational bands, $0\,\mathrm{cm}^{-1}$, $1600\,\mathrm{cm}^{-1}$, and $3900\,\mathrm{cm}^{-1}$. As also shown in Fig. 6**b**–**c** of the main text, these correspond to the bending and stretching modes of water, with the bending modes centred near 1600 cm^{-1} and the OH stretching modes near 3900 cm^{-1}. The stretching frequency in particular is somewhat higher than typically observed in experiment and we have therefore also considered the vibrational power spectrum of an isolated water molecule in vacuum,

as shown in Supplementary Figure **15**.

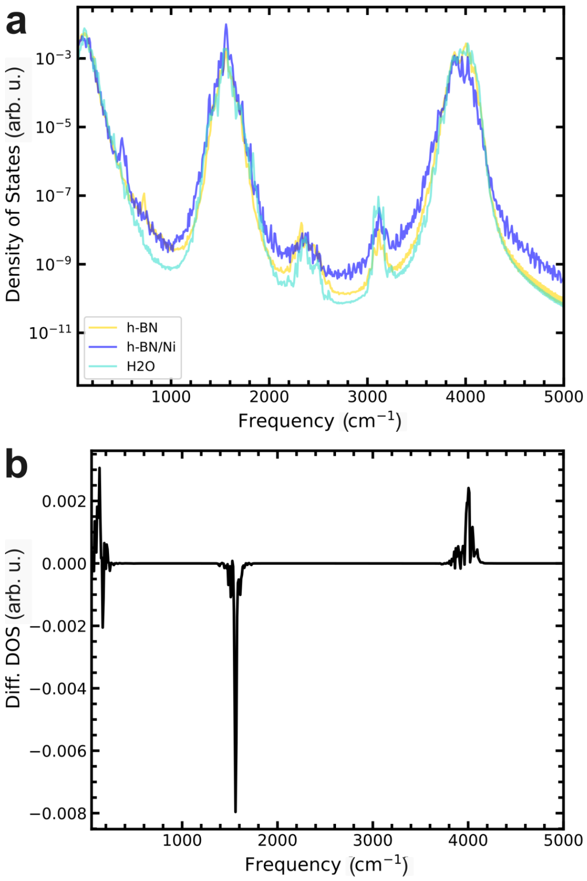

Supplementary Figure 14: **Comparison of vibrational spectra across h-BN and h-BN/Ni.** **a** The ensemble average of the power spectrum of the velocity autocorrelation function for different conditions: h-BN, h-BN/Ni, h-BN with the dimeric water system, and isolated water. The spectrum is derived from ab initio molecular dynamics trajectories. Three clear bands corresponding to low-velocity group motion and inter- and intra-molecular vibrations are observed. **b** Replotting the spectrum but showing the difference in the vibrational bands between the free-standing h-BN and h-BN with the Ni substrate.

The frequencies exhibit a stretch near 3900 cm^{-1}, consistent with the results of water on a surface, which suggests that the observed redshift for interfacial water is not primarily due to molecule–surface interactions. Instead, the elevated stretching frequencies are a known artifact of DFT-based harmonic vibrational analysis. In particular, OH stretching modes are commonly overestimated by 8–12% due to the neglect of anharmonicity and limitations of the exchange–correlation functional. Our computed value around 3900 cm^{-1} is thus higher than the experimental OH stretch values, which are typically found near 3750 cm^{-1}. This is consistent with recent benchmark studies, including Refs[19,20], which highlights that DFT overestimates C–H, N–H, and O–H stretching frequencies by over 10%, and that empirical scaling factors are often employed to correct for this discrepancy. However, in our analysis we focus on relative shifts rather than absolute frequencies, comparing, for example, the OH stretch with and without the supporting metal substrate. These relative differences are more robust, as many of the systematic errors cancel out.
The most prominent peak in all cases is the 0 cm^{-1} corresponding to the small group velocity of the water tumbling along the surface. The peak near the origin for dimeric systems has a longer high-frequency tail than the monomeric systems; this likely corresponds to the intermolecular modes between the waters. The intermolecular librational, stretching, and bending motions are concentrated into the low-frequency peak that appears in the 50–800 cm^{-1} range. On the other hand, the monomeric cases have a slightly higher value but are devoid of extra low-frequency peaks. A large band of vibrations is peaked at 1600 cm^{-1}, corresponding to the H–O–H bending motion. The peak exhibits the same broadness for both the monomeric and dimeric systems but is an order of magnitude stronger for the dimeric system. The 3900 cm^{-1} band comprises several O–H stretching modes. A smaller extra peak is also observed at 3150 cm^{-1} for the dimer system, which is suppressed in the monomer case.

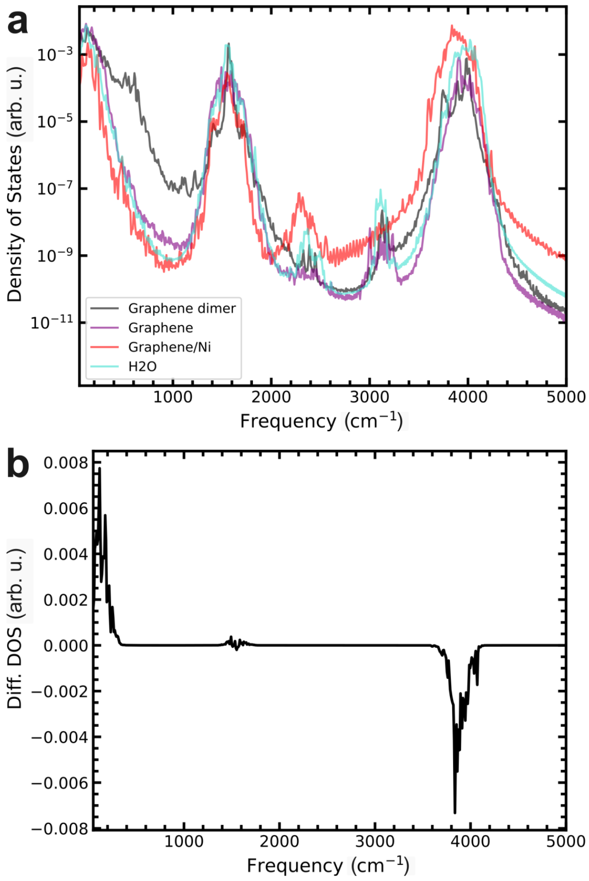

Supplementary Figure 15: **Comparison of vibrational spectra across graphene and graphene/Ni.** **a** The ensemble average of the power spectrum of the velocity autocorrelation function for different conditions: graphene, graphene with a Ni substrate, graphene with the dimeric water system, and isolated water. The spectrum is derived from ab initio molecular dynamics trajectories. Three clear bands corresponding to low-velocity group motion and inter- and intra-molecular vibrations are observed. **b** Replotting the spectrum but showing the difference in the vibrational bands between the free-standing h-BN and h-BN with the Ni substrate.

Supplementary Figure **14** compares the change in the vibrational power spectrum of the free-standing vs the Ni-supported surface. The band at 1600 cm^{-1} shows the largest induced changes with a significant dip, whereas some minor changes are seen in the 3900 cm^{-1} band. On the one hand, graphene follows a similar trend to h-BN. The same three bands appear at 0 cm^{-1}, 1600 cm^{-1}, and 3900 cm^{-1}. In the dimer case, the 0 cm^{-1} band is comparatively broadened, whereas the 1600 cm^{-1} band is much weaker in the monomer case. Overall, more spectral weight is distributed into the 1600 cm^{-1} and 3900 cm^{-1} bands for the dimeric

system, while the $0\,\mathrm{cm}^{-1}$ band remains more pronounced in the monomeric case.
On the other hand, introducing the Ni substrate suppresses certain modes within the $0\,\mathrm{cm}^{-1}$ band and leads to only minor changes around $1600\,\mathrm{cm}^{-1}$, while the $3900\,\mathrm{cm}^{-1}$ band becomes noticeably stronger in the presence of Ni.

2.12 Electronic density of states

The electronic density of states (DOS) was calculated using the OptaDOS code[21], and the partial DOS plots for C (graphene) and B and N (h-BN) can be seen in Supplementary Figure **16**. The k-points mesh was increased to $12 \times 12 \times 1$ for these calculations. As expected, the plots for the free-standing material show a zero band gap for graphene and an insulator character for free-standing h-BN.

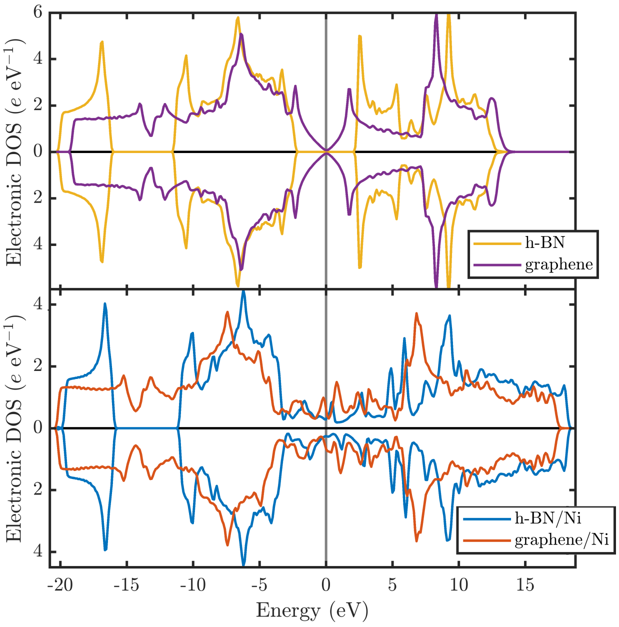

Supplementary Figure 16: Partial electronic density of states (PDOS) for the top-most layer of the free-standing and Ni-supported graphene and h-BN systems. The spin-up and spin-down components are shown in the upper and lower halves of each plot, respectively. The Fermi level (grey vertical line) was shifted to 0 for better clarity of the plots. As can be seen in the lower plot, upon inclusion of the Ni(111) substrate, both h-BN and graphene exhibit a similar DOS at and around the Fermi level (E_{F}). Thus, the electronic structure does not exhibit a strong impact on the friction, and we also expect that (non-adiabatic) electron-hole pair excitations are possible in both cases, giving rise to a comparable contribution to electronic friction on both h-BN/Ni and graphene/Ni.

The presence of the Ni support introduces states near the Fermi level, illustrated by the grey vertical line. The Fermi level (E_{F}) itself is shifted by $1.60\,\mathrm{eV}$ and $0.89\,\mathrm{eV}$ when including the Ni support for graphene and h-BN, respectively. The graphene/Ni(111) system has a density of states ca. 2.5 times higher ($1.29\,e\,\mathrm{eV}^{-1}$ for graphene/Ni and $0.53\,e\,\mathrm{eV}^{-1}$ for hBN/Ni, accounting spin up and spin down contributions) at the Fermi level compared to h-BN/Ni(111). Due to the small differences observed in the electronic DOS, the large friction observed on graphene/Ni(111) is not a consequence of the electronic structure of the substrate but rather a combination of the corrugation of the water energy landscape and vibrational effects.

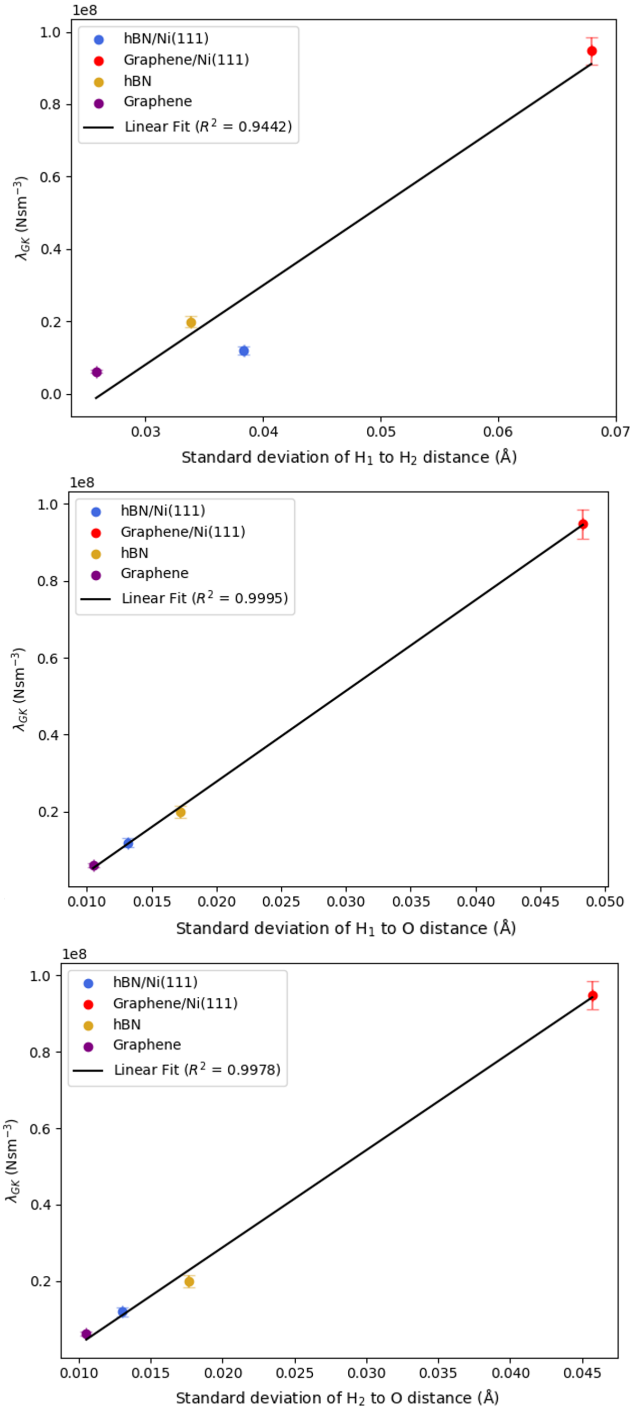

Supplementary Figure 17: Standard deviation of the H–H, $H_{(1)}$–O, and $H_{(2)}$–O bond distances experienced by the water molecule in the NVE simulations. In each case the high values for R^2 indicate a strong correlation between vibrational modes and λ_{GK}. Error bars representing the margin of error (corresponding to the 98% confidence interval to the mean) from 10 independent NVE simulations per system.

2.13 Regression and temporal evolution

We employed linear regression analysis to uncover the intricate relationship between the friction coefficient λ_{GK} (calculated using the Green-Kubo relationship) and the dynamic behaviour of water molecules. Our investigation encompassed an exploration of the correlation between λ_{GK} and various physical parameters. A normal distribution was used to calculate the z-score (Z), and

the margin of error (ME) was calculated using the formula:

$$\mathrm{ME} = Z \cdot \left(\frac{\sigma}{\sqrt{n}}\right) \quad (3)$$

Where σ is the standard deviation and n is the number of samples. Simulations were divided into 2 ps segments, λ_{GK}, ΔG, H-H and H-O distances were calculated for each 2 ps segment averaging across all segments to find the mean for each system. In all cases shown in Fig. 6**d** in the manuscript, the models exhibit a positive correlation, supported by statistically significant differences as assessed by one-way ANOVA[22] ($p < 0.01$).

Supplementary references

1. Auwärter, W., Kreutz, T., Greber, T. & Osterwalder, J. XPD and STM investigation of hexagonal boron nitride on Ni(111). *Surf. Sci.* **429**, 229–236 (1999).
2. Ruckhofer, A. *et al.* Evolution of ordered nanoporous phases during h-BN growth: Controlling the route from gas-phase precursor to 2D material by in-situ monitoring. *Nanoscale Horiz.* **7**, 1388–1396 (2022).
3. Attard, G. *Surfaces*. Oxford Chemistry Primers (Oxford Univeristy Press, Oxford, 1998).
4. Chakarov, D. V., Oesterlund, L. & Kasemo, B. Water adsorption and coadsorption with potassium on graphite(0001). *Langmuir* **11**, 1201–1214 (1995).
5. Chakarov, D., Österlund, L. & Kasemo, B. Water adsorption on graphite (0001). *Vacuum* **46**, 1109–1112 (1995).
6. Bolina, A. S., Wolff, A. J. & Brown, W. A. Reflection Absorption Infrared Spectroscopy and Temperature-Programmed Desorption Studies of the Adsorption and Desorption of Amorphous and Crystalline Water on a Graphite Surface. *J. Phys. Chem. B.* **109**, 16836–16845 (2005).
7. Tamtögl, A. *et al.* Motion of water monomers reveals a kinetic barrier to ice nucleation on graphene. *Nat. Commun.* **12**, 1–8 (2021).
8. Ward, D. J. *A Study of Spin-echo Lineshapes in Helium Atom Scattering from Adsorbates*. Ph.D. thesis, University of Cambridge (2013).
9. Glebov, A., Graham, A. & Menzel, A. Vibrational spectroscopy of water molecules on Pt(111) at submonolayer coverages. *Surf. Sci.* **427**, 22–26 (1999).
10. Poelsema, B. & Comsa, G. *Scattering of Thermal Energy Atoms*, vol. 115 of *Springer Tracts in Modern Physics* (Springer Berlin Heidelberg, 1989).
11. Farias, D. & Rieder, K.-H. Atomic beam diffraction from solid surfaces. *Rep. Prog. Phys.* **61**, 1575–1664 (1998).
12. Redhead, P. A. Thermal desorption of gases. *Vacuum* **12**, 203–211 (1962).
13. Ulbricht, H., Zacharia, R., Cindir, N. & Hertel, T. Thermal desorption of gases and solvents from graphite and carbon nanotube surfaces. *Carbon* **44**, 2931–2942 (2006).
14. Smith, R. S., Matthiesen, J. & Kay, B. D. Desorption kinetics of methanol, ethanol, and water from graphene. *J. Phys. Chem. A* **118**, 8242–8250 (2014).
15. Belyaeva, L. A., Tang, C., Juurlink, L. & Schneider, G. F. Macroscopic and microscopic wettability of graphene. *Langmuir* **37**, 4049–4055 (2021).
16. Nørskov, J. K., Studt, F., Abild-Pedersen, F. & Bligaard, T. *Fundamental concepts in heterogeneous catalysis* (John Wiley & Sons, 2014).
17. Avidor, N. *et al.* Pigle — particles interacting in generalized langevin equation simulator. *Comput. Phys. Commun.* **242**, 145–152 (2019).
18. Tocci, G., Joly, L. & Michaelides, A. Friction of Water on Graphene and Hexagonal Boron Nitride from *Ab Initio* Methods: Very Different Slippage Despite Very Similar Interface Structures. *Nano Lett.* **14**, 6872–6877 (2014).
19. Varandas, A. J. C. Scale-free-modeling (harmonic) vibrational frequencies: Assessing accuracy and cost-effectiveness by CBS extrapolation. *J. Chem. Phys.* **157**, 174110 (2022).
20. Medel, R. & Suhm, M. A. Predicting OH stretching fundamental wavenumbers of alcohols for conformational assignment: different correction patterns for density functional and wave-function-based methods. *Phys. Chem. Chem. Phys.* **23**, 5629–5643 (2021).
21. Morris, A. J., Nicholls, R. J., Pickard, C. J. & Yates, J. R. Optados: A tool for obtaining density of states, core-level and optical spectra from electronic structure codes. *Comput. Phys. Commun.* **185**, 1477–1485 (2014).
22. Girden, E. R. *ANOVA: Repeated measures*. 84 (Sage, 1992).
