# Supplementary material for: Understanding water behaviour on 2D material interfaces through single-molecule motion on h-BN and graphene
Source: Nat Commun. 2025 Nov 25;16:10465. doi: 10.1038/s41467-025-65452-1 (PMC12647898; doi:10.1038/s41467-025-65452-1)
Supplement: Supplementary file 2 — Description of Additional Supplementary Files [file 41467_2025_65452_MOESM2_ESM.pdf]

Description of Additional Supplementary Files

File Name: Supplementary Movie 1

Description: Ab initiomolecular dynamics (AIMD) simulation showing the motion of a single water molecule on a hexagonal boron nitride (h-BN) monolayer supported on a Ni(111) surface. The top panel presents a side view, revealing the vertical fluctuations and adsorption geometry of the molecule relative to the surface. The bottom panel shows a top view, highlighting the lateral diffusion pathway. The movie illustrates not only translational motion but also the characteristic rotational and precessional dynamics of the water molecule as it traverses the h-BN/Ni(111) surface.

File Name: Supplementary Movie 2

Description: Ab initio molecular dynamics simulation of a single water molecule on hBN/Ni(111). The top panel shows a side view, illustrating the adsorption geometry and vertical motion. The bottom panel shows a top view, capturing the molecule's lateral diffusion along with its rotational and precessional dynamics.

File Name: Supplementary Movie 3

Description: Ab initio molecular dynamics simulation of a single water molecule on hBN/Ni(111). The top panel shows a side view, illustrating the adsorption geometry and vertical motion. The bottom panel shows a top view, capturing the molecule's lateral diffusion along with its rotational and precessional dynamics.
